# Long-term follow-up and treatment of congenital alveolar proteinosis

**DOI:** 10.1186/1471-2431-11-72

**Published:** 2011-08-17

**Authors:** Matthias Griese, Jan Ripper, Anke Sibbersen, Pia Lohse, Peter Lohse, Frank Brasch, Andrea Schams, Asli Pamir, Bianca Schaub, Oliver J Muensterer, Carola Schön, Judith Glöckner-Pagel, Thomas Nicolai, Karl Reiter, Andreas Hector

**Affiliations:** 1 Dr. von Haunersches Kinderspital, University of Munich, Munich, Germany; 2 Department of Clinical Chemistry-Großhadern, University of Munich, Munich, Germany; 3 Institute for Pathology, Bielefeld, Germany; 4 Weill-Cornell Medical Center, Division of Pediatric Surgery, New York, NY, USA

**Keywords:** pulmonary alveolar proteinosis, therapeutic lung lavage, GM-CSF-R alpha, genetic defect, stop codon

## Abstract

**Background:**

Clinical presentation, diagnosis, management and outcome of molecularly defined congenital pulmonary alveolar proteinosis (PAP) due to mutations in the GM-CSF receptor are not well known.

**Case presentation:**

A 2 1/2 years old girl was diagnosed as having alveolar proteinosis. Whole lung lavages were performed with a new catheter balloon technique, feasible in small sized airways. Because of some interstitial inflammation in the lung biopsy and to further improve the condition, empirical therapy with systemic steroids and azathioprin, and inhaled and subcutaneous GMCSF, were used. Based on clinical measures, total protein and lipid recovered by whole lung lavages, all these treatments were without benefit. Conversely, severe respiratory viral infections and an invasive aspergillosis with aspergilloma formation occurred. Recently the novel homozygous stop mutation p.Ser25X of the GMCSF receptor alpha chain was identified in the patient. This mutation leads to a lack of functional GMCSF receptor and a reduced response to GMCSF stimulation of CD11b expression of mononuclear cells of the patient. Subsequently a very intense treatment with monthly lavages was initiated, resulting for the first time in complete resolution of partial respiratory insufficiency and a significant improvement of the overall somato-psychosocial condition of the child.

**Conclusions:**

The long term management from early childhood into young adolescence of severe alveolar proteinosis due to GMCSF receptor deficiency requires a dedicated specialized team to perform technically demanding whole lung lavages and cope with complications.

## Background

Pulmonary alveolar proteinosis (PAP) is characterized by a substantial and persistent increase in surfactant pool size [1,2]. There are several causes of this rare condition; mouse models with deletion of granulocyte-macrophage-colony stimulating factor (GM-CSF) or the GM-CSF receptor (GM-CSFR) beta-chain showed the first evidence for involved molecularly mechanisms [3,4]. Autoantibodies against GM-CSF, blocking GM-CSF signaling, are the cause for the most frequent form of PAP, mainly found in adults and also called autoimmune PAP [5]. In 2008 the first two families with congenital PAP and mutations in the alpha-chain of the receptor for GM-CSF were described [6,7] and very recently another six families were reported [8]. The patients presented with progressive dyspnea of insidious onset between the ages of 1.5 and 9 years; some were asymptomatic. Short term responsiveness to whole lung lavage (WLL) treatment has been described, however not much information on the long term outcome of molecularly defined patients is yet available.

Other genetic conditions that lead to PAP include a recently identified mutation in the beta chain of the GMCSF receptor [9], surfactant protein B or C deficiency [10,11], Niemann-Pick Type C2 disease [12] and lysinuric protein intolerance [13]. Secondary PAP develops in association with conditions involving functional impairment or reduced numbers of alveolar macrophages like inhalation of inorganic dusts, myeloic leukemia, myelodysplastic syndrome, immunosuppression related to organ transplantation, and some infections including Pneumocystis [1].

Not much is known on the clinical spectrum, course and treatment options in patients with molecularly defined, congenital PAP due to mutations in the GM-CSF alpha chain. Also the role of long term WLL, which are be very demanding due to the small size of the airways, how to measure clinical response to lavage therapy and the relevance of glucocorticoid therapy have not been reported. Here we present the successful management of a child with a severe congenital PAP caused by the homozygous p.Ser25X mutation in exon 3 of the CSF2RA gene and its follow up for more than a decade. These data may be helpful for future treatment of infants and children with this rare condition.

## Case Presentation

**Figure 1:**
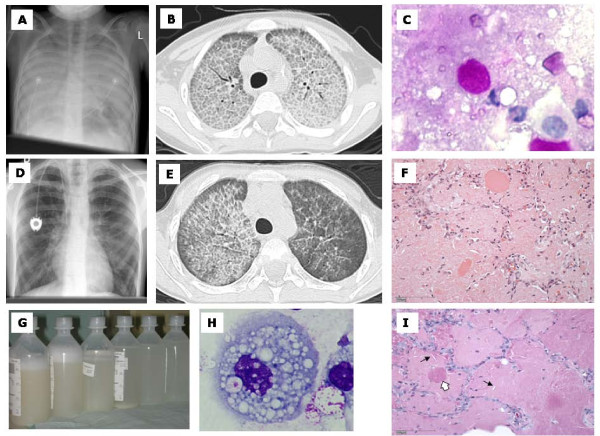
**Diagnosis of alveolar proteinosis was suspected based on typical radiological picture in chest x-ray and CT with ground-glass attenuation and interstitial thickening, resulting in crazy paving pattern (Figure. 1 A, B)**. Figure. 1 E shows CT after whole lung lavage of the right lung. Macroscopic appearance (Figure. 1 G) and light microscopy of BAL fluid stained with PAS (Figure. 1 C, magnification x400) showed extracellular positive proteinaceous material, lipid-laden macrophages (Figure. 1 H, MGG stain, magnification x1000), and after exclusion of infectious or metabolic causes or malignancy, PAP was confirmed by histology (right lower lobe). HE stained tissue demonstrated alveolar filling with eosinophilic material (Figure. 1 F) which was positive in periodic acid stain (PAS)(Figure. 1 I), intraalveolar cholesterol clefts (Figure. 1 H, arrows) and characteristic oval bodies (Figure. 1 H, open arrow).

The patient was the 2nd of 3 living children; born at term in 1998, with no immediate postnatal respiratory distress. The family history was unremarkable for pulmonary or other rare diseases; the parents were consanguineous and of Turkish descent. At age 2 1/2 years during an acute respiratory tract infection with productive cough and fever with no response to antibiotics, intermittent cyanosis occurred and the child was referred to our centre because of chronic tachypnoea and weight loss. Because of a typical chest computed tomography (CT) and chest x-ray (Figure 1A, B), BAL macroscopic appearance (Figure 1G) and microscopy (Figure 1C, H), and after exclusion of infectious or metabolic causes or malignancy, PAP was suspected and confirmed by histology (Figure 1F, I).

**Figure 2:**
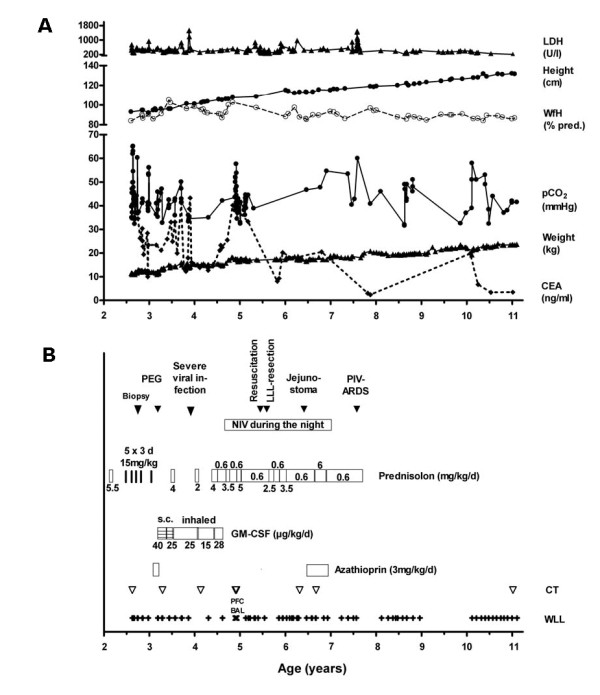
**Long term clinical course and treatments**. Lactate dehydrogenase (LDH), height, weight for height (WfH) expressed as the current weight of the child as a percentage of the normal weight for a given height, capillary CO2 pressure, and carcino embryonic antigen, CEA in serum (Figure. 2 A). For nutrition a percutaneous gastrostomy (PEG) and later a jejunostoma were implemented. Whole lung lavages (WLL) were performed as indicated; at age 5 years four consecutive lavages were done with perfluorocarbon (PFC BAL). Further abbreviations: ARDS: acute respiratory distress syndrome, LLL: left lower lobe, NIV: non invasive nasal-mask ventilation, PIV: Parainfluenza virus (Figure. 2 B).

At presentation the child had global respiratory insufficiency, combined with an elevated level of LDH and CEA (Figure 2A). Based on the clinical diagnosis of alveolar proteinosis we initiated whole lung lavages and some additional treatments.

**Figure 3:**
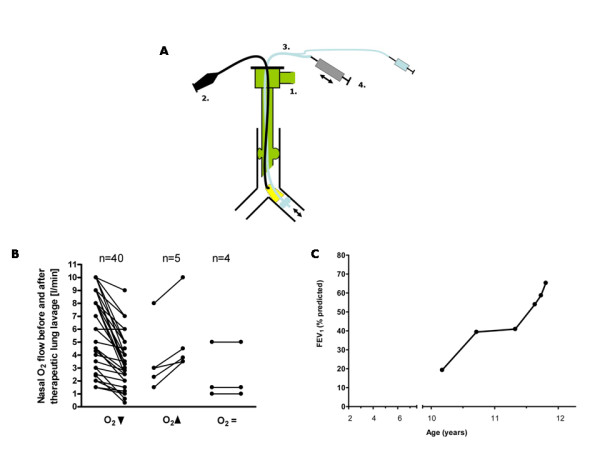
**Whole lung lavages were performed, due to the small sizes of the airways until the age of 6 y, via a blocked endotracheal tube through which the child was ventilated (Figure. 3 A, 1.) and through which also a pulmonary artery catheter (Figure. 3 A, 3., blue) was inserted and blocked in the left or right main stem bronchus (small syringe) and the lavage was done (Figure. 3 A, 4., large syringe)**. The tight fit of the blocked pulmonary artery catheter was continuously monitored via a 1.8 mm endoscope (Figure. 3 A, 2., black). Figure. 3 B shows the change of nasal flow of oxygen before and after the first 49 whole lung lavages (P < 0.0001, paired comparison by Wilcoxon test); in the figure values are ordered according observed change in oxygen flow ("triangle tip down" indicates reduced flow after lavage, "triangle tip upwards" indicates increased flow of oxygen after lavage, and " = " indicates unchanged flow of oxygen. From year 10 onward lung function tests were performed, forced vital capacity (FVC) improved dramatically with more intensely performed lavages (not shown), as did forced expiratory volume in 1 second (FEV1) (Figure. 3 C).

**Figure 4:**
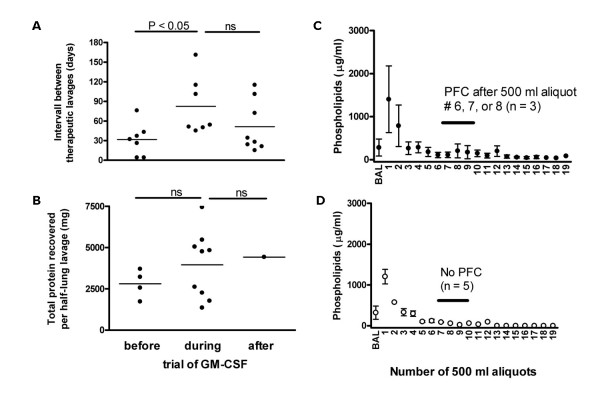
**At age 5 y, during four consecutive whole lung lavage sessions perfluorocarbon (PFC) was instilled into the lungs after the 6th, 7th, or 8th of the 500 ml wash in order to enhance the recovery of surfactant material**. The concentration of phosphoplipids (and total protein (not shown)) washed out was not significantly altered compared to lavages done at that age without PFC (Figure. 4 A, B). From age 3 to 4 1/2 years therapy with inhaled and subcutaneous injections of recombinant GM-CSF was done (see also Figure. 2 B). Although the intervals between consecutive therapeutic whole lung lavages were increased with GM-CSF treatment the amount of protein removed was also increased, demonstrating no reduction of protein amount with GM-CSF treatment (Figure. 4 C, D). Note that not all lavages were available for total protein measurements.

Therapeutic bronchoalveolar lavages of either the right or left lung were performed under general anaesthesia and paralysis. From age 2 1/2 to 6 years we used a technique that isolated one lung with the help of a balloon catheter (5-7 Fr, Arrow, Reading, USA) placed into one main stem bronchus through the cuffed endotracheal tube (ID 4.0-5.0) and blocked there (see also Figure 3A). The position and fitting of the catheter was permanently monitored via an Olympus bronchoscope (BFN20, O.D. 1.8 mm), as described in detail [14]. From age 7 onward the size of the main bronchus was sufficient to allow double lumen tube and lavage of one lung, while ventilating the other. Sterile 0.9% NaCl warmed to body temperature was used as lavage fluid. The lavage was done by manual injection and withdrawal of saline from a 50 ml syringe, starting with smaller volumes of 4 ml/kg body weight (used for diagnostic purposes) and increasing to about 11 to 15 ml/kg under continuous control of the correct position and tightness of the balloon. The recovered fluid was collected via a 2-way cock stop into 500 ml bottles to allow judgement of turbidity and to follow the process of lavage [15]. In 2003, at age 5 y, we tried to increase the yield of lipo-proteins recovered from the lungs with the help of perfluorocarbons (PFC) [16,17]. A total of 100 ml of Perfourodecalin (Pharmpur, Augsburg, Germany) was instilled in 3 aliquots after the 6th, 7th, or 8th 500 ml wash during three consecutive WLL sessions. This approach did neither improve the yield of phospholipids washed out (Figure 4A, B) nor that of total protein (not shown).

Empirically, we found that WLL were the most efficient treatment. This was clearly shown for the short term; during 49 instances investigated until the age of 11, the amount of nasal oxygen flow was reduced in 40 after the lavages (Figure 3B). This effect could be sustained for many years demonstrating long term efficacy. However the extreme value of WLL was only very recently demonstrated unequivocally following implementation of our concept of very aggressive WLL. Up to the age of 10 years, WLL were done more or less to ameliorate partial respiratory insufficiency, i.e. to decrease the need for additional oxygen. From year 10 onward, we performed one lavage per month, in order to try to completely clear the lung from its proteinosis load. This approach was very successful and resulted in complete resolution of partial respiratory insufficiency for the first time. The patient started puberty, growth and weight were sustained by oral nutrition without need of using the percutaneous tube and the dependency on supplemental oxygen up to that point in time, could be finished. This also led to increased self-confidence and better integration at school. Also, the lung function improved very rapidly and chest radiograph cleared to almost normal (Figure 1D, E; Figure 3C). Together somato-psychosocial condition substantially improved. A consecutive brief trial to increase the time lag between the lavages failed and an interval of about 4 weeks was maintained.

**Figure 5:**
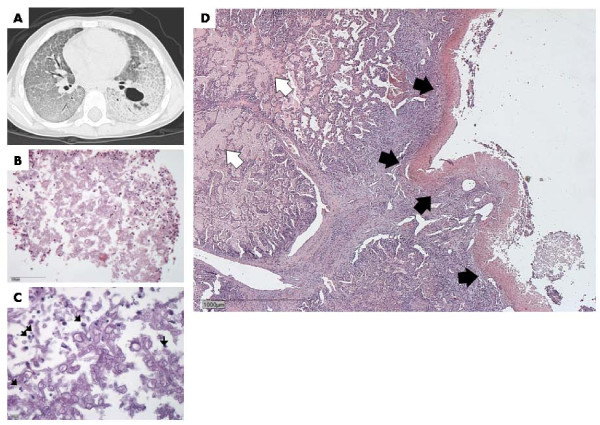
**Clinical complication**. At age 5 years a chest CT (Figure. 5 A) showed a cavity and infiltration in the left lower lobe which was due to an infection with Aspergillus fumigatus; the resected specimen showed an aspergilloma (black arrows) in the neighborhood of typical alveolar proteinosis with surfactant filled alveoli (white arrows)(Figure. 5 D). Inside the cavity a neutrophilic infiltrate (Figure. 5 B) and infection with aspergillus (Figure. 5 C, almost all fungi, arrows indicate neutrophils) was present.

Because at clinical diagnosis of the patient, both the exact cause of the PAP and effective treatments in small children were unknown, empirical high dose glucocorticosteroids in pulses were used (Figure 2A) and under the impression that they might be helpful, systemic corticosteroids were used for prolonged periods until the age of 7.5 years. During this time, azathioprine as a steroid saving agent was also used without any apparent benefit. However, we clearly observed that severe infectious complications were only observed during the time of increased immunosuppression by these agents (Figure 2A, B). At 5 years of age an Aspergillus fumigatus infection with formation of a cavity, leading to severe cardio-respiratory failure and resuscitation followed by resection of the left lower lobe (Figure 2B, Figure 5A-D). At age 7.5 years she suffered a pulmonary para-influenza infection, leading to ARDS and necessitating mechanical ventilation. Additionally, the child had many mild respiratory exacerbations, mostly believed to be induced by viral upper- and lower respiratory tract infections.

Of interest, from age 3 to 4 1/2 years, well before the molecular nature of the PAP was determined, we used inhaled and subcutaneous recombinant GM-CSF (Figure 2B). A transient increase in peripheral blood eosinophils up to 17% of the neutrophils occurred [18] (data not shown), but clearly no improvement of the alveolar proteinosis (Figure 2A, B, Figure 4C, D). Due to the expectations of the treating physicians, the intervals between consecutive therapeutic WLL were increased during GM-CSF treatment: In parallel, this was associated with an increased load of protein, demonstrating a lack of an effect of GM-CSF treatment (Figure 4C, D).

Nutritional support was optimized with the help of a percutaneous gastrostomy (PEG, Figure 2B) placed at the age of 3 years, which was used regularly; the gastrostomy was changed to a jejunostoma at the age of 61/2 years, to completely exclude gastro-esophageal refluxes, although no such events had been demonstrated in pH- or impedance studies (Figure 2B).

### GM-CSF, the GM-CSF receptors and their functional analysis

**Figure 6:**
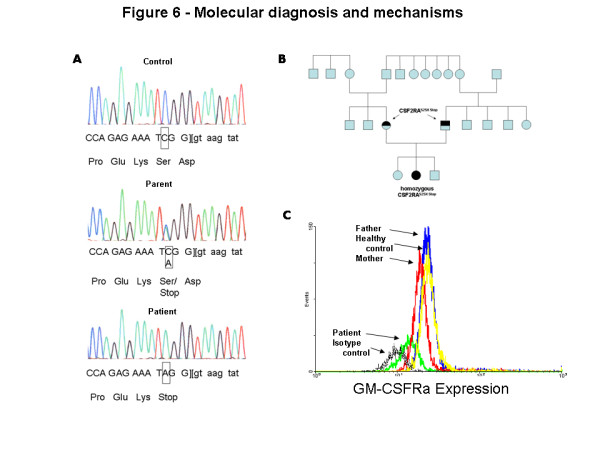
**Molecular diagnosis and mechanism**. Sequence analysis of CSF2RA exon 3 in a control subject (Figure. 6 A, upper panel), in one of the parents (Figure. 6 A, middle panel), and in the proband (Figure. 6 A, lower panel). In peripheral blood leukocytes genomic DNA, exons 3-13 of the CSF2RA gene were amplified and the PCR products were sequenced. The electropherograms illustrate the c.74 C > A substitution (boxed) in heterozygous (middle panel) and in homozygous form (lower panel), which leads to the replacement of a serine (TCG) by a premature stop codon (TAG) at amino acid position 25. Family tree shows consanguinity (Figure. 6 B). Flow cytometric analysis of peripheral blood cells demonstrates the absence of the alpha-chain (Figure. 6 C). Isolated neutrophils were incubated with antibodies, washed with Dulbecco's PBS twice and measured by means of flow cytometry. Ten thousand cells were counted and analyzed by the FACSDiva software. Fc blocking and isotype controls were applied to exclude unspecific bindings. CD11b (mouse monoclonal IgG1, PE conjugated), CD116/GM-CSF-R alpha (mouse monoclonal IgG1, PE conjugated), CD131w/GM-CSF-R beta (mouse monoclonal IgG1, purified) and secondary antibody for CD131w (rat anti-mouse IgG1, PE-conjugated).

At age 12 years (in 2009) analysis of the patient's *CSF2RA *gene revealed the homozygous Ser25X stop-mutation in exon 3 resulting in the almost complete absence of the GM-CSF receptor alpha chain and causing the alveolar proteinosis we observed (Figure 6A). The parents were heterozygous for the mutation (Figure 6A, B). Mutations in SFTPC, SFTPB and ABCA3 were excluded.

**Figure 7:**
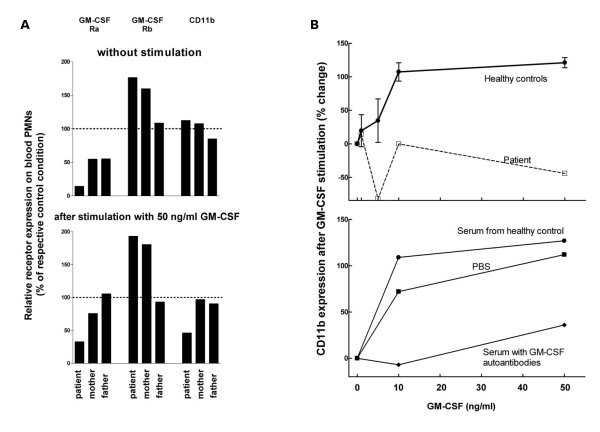
**Molecular diagnosis and mechanism**. Peripheral neutrophils were isolated by means of density-gradient centrifugation with Ficoll (GE Healthcare, Munich, Germany and assessed in the presence and absence of GM-CSF for the expression of the GM-CSF Ra and Rb chain, and CD11b. The purity of neutrophils was greater than 95% as assessed by differential cell counts of Pappenheim cytospin preparations. Cell viability was greater than 95% using trypan-blue exclusion method. 106 neutrophils were treated with 50 ng/ml GM-CSF or the same volume of the vehicle buffer (aqua a.i.) for 30 min at 37°C. In the absence of GM-CSF stimulation, GM-CSF-Ra chain expression in the parents was only 50% of that of the controls (Figure. 3 A), whereas GM-CSF-Rb chain and CD11b expression were normal. After stimulation with GM-CSF, GM-CSF-Ra in the patient remained low, whereas the parents' level increased to almost control level. GM-CSF-Rb chain and CD11b stimulation were normal (Figure. 3 A). CD11b expression on the neutrophils was used to assess signal transduction after GM-CSF stimulation. In the patient, similar as blocked by auto-antibodies from a subject with auto-immune PAP, no dose-dependent stimulation of CD11b was observed, demonstrating interruption of signalling (Figure. 3 B).

GM-CSF level were increased in serum (106 pg/ml, normal < 6 pg/ml) of the child and normal in the parents. No anti-GM-CSF autoantibodies were detected in serum [19]. GM-CSF-Ra chain expression after stimulation with 50 ng/ml GM-CSF on peripheral mononuclear cells of the patient was markedly reduced and normal in both parents (Figure 6C). In the absence of GM-CSF stimulation, GM-CSF-Ra chain in the parents was only 50% of that of the controls (Figure 7A), whereas GM-CSF-Rb chain and CD11b were normal. After stimulation with GM-CSF, GM-CSF-Ra and CD11b remained low in the patient (Figure 7A, lower panel), whereas the parents' levels were in the normal range level. GM-CSF-Rb chain without and with GM-CSF stimulation was normal (Figure 7A). CD11b expression on the neutrophils was used to assess signal transduction after GM-CSF stimulation. In the patient, similar as blocked by auto-antibodies from a subject with auto-immune PAP, no dose-dependent stimulation of CD11b was observed, demonstrating interruption of signalling (Figure 7B).

## Conclusions

Here we report a patient with molecularly defined severe congenital PAP due to a previously undescribed autosomal recessive mutation in the alpha chain of the GM-CSF receptor. This mutation leads to a stop of transcription and to a lack of functional protein. The GM-CSF induced responses are mediated through activation of the transcription factor PU.1 and include increased surfactant catabolism and CD11b expression [20]. Impairment of the latter was shown directly in mononuclear cells of the patient after stimulation with GM-CSF. Impaired GMCSF receptor activation of alveolar macrophages leads to decreased surfactant catabolism and accumulation of surfactant in the alveolar space, i.e. alveolar proteinosis.

Important messages from this study are related to the long-term management of this condition. First, persistent and aggressive removal of surfactant filling the alveolar space may eliminate gas exchange abnormalities and consecutive sequelae including developmental and growth failure, and restricted level of performance due to respiratory limitation. Second, immune insufficiency, a problem also primarily resulting from abnormalities of the GM-CSF signal transduction pathway [20], may be augmented by immunosuppressive therapy initiated to treat the condition empirically. Therefore, molecular genetic definition of the basic defect in all children with PAP is important. Lastly, we describe the successful use of outcome measures of the efficacy of therapeutic WLL, including oxygen demand, and amount of washed out protein and phospholipids.

A major strength of this study is to demonstrate the feasibility of technically demanding repetitive WLL in a very small child over extended periods of time. Although therapeutic WLL is generally accepted as the established treatment option for PAP in adults, its optimal method, frequency of application and many other details are currently not known in infants or children. Here we show that consecutive lavages via a small catheter located in a main stem bronchus (Figure 3A) can be used to efficiently remove accumulated surfactant from the alveolar space in a very small child. Furthermore we show that it is helpful to monitor efficacy of the washing procedure by determination of proteins and lipids removed from the lungs [15]. These measurements allowed us to demonstrate only a marginal, but not clinically significant increase in the removal of surfactant material from the lungs, by the use of PFC for lavage. In a case report on an infant with alveolar proteinosis due Niemann Pick disease the usage of PFC was recently shown not to be of benefit as well [21].

Although feasibility of the long term management of congenital PAP with WLL was demonstrated in this case of severe PAP, molecular diagnosing PAP as caused by a genetic deficiency of GM-CSFRa may have other important prophylactic and therapeutic implications. First, based on experiments in mice with PAP bone marrow transplantation may cure the disease [3]. Currently we believe however that the risks of a bone marrow transplant (chronic graft versus host disease, among others) outweigh its benefits (elimination of need for WLL). Second, if diagnosed early in a family with an index case, the opportunity of early intervention by lavages at times of good clinical condition will help to reduce complications.

Subcutaneous injections or inhalations of GM-CSF, which have been successfully utilized in adult patients with autoimmune PAP [22,23], were not helpful in our case to reduce alveolar filling as assessed by CT scanning (not shown) or improvement in gas exchange (Figure 2). Treatment with 20 μg/kg of GM-CSF per day subcutaneously was also shown to be ineffective for the child with congenital PAP described by Martinez-Moczygemba et al. [7].

Immunosuppressive treatment was used empirically and because of the presence of neutrophils and some lymphocytes in the lavage specimens and in the interstitial space of the lung biopsy sample of the patient (Figure 2B). Unfortunately severe and prolonged infections occurred, including a cavity forming infection with Aspergillus fumigatus which was treated by i.v. and inhaled amphotericin B and surgical resection of the cavity. Sustained withdrawal of the systemic corticosteroids from age 8 years onward did not alter the activity of the underlying PAP, but reduced the rate of infectious respiratory complications considerably.

Our study exemplifies detailed long term management of severe molecularly defined alveolar proteinosis from childhood into young adolescence. It is of interest that a dedicated specialized team may be advantageous to maintain the appropriate expertise of complex procedures such as e.g. whole lung lavages in small children [8,15,24,25]. Therefore a centralized approach, as it has been employed for rare lung diseases and PAP in particular on a national basis in France [24], may be warranted. A web-based system to collect these rare cases, follow them and also to receive support is available at the kids lung register (http://www.kids-lung-register.eu). The novel whole lung lavages technique using an inflatable balloon catheter was feasible in very small sized airways. Whereas empirical immunosuppressive therapy and inhaled and subcutaneous GMCSF were without significant benefit, a very intense treatment with WLL resulted in complete resolution of respiratory insufficiency, and a normalisation of lung physiology and overall somato-psychosocial condition of the child.

## Abbreviations

PAP: Pulmonary alveolar proteinosis; GMCSF: granulocyte-macrophage-colony stimulating factor; GM-CSFR: GM-CSF receptor; WLL: whole lung lavage; washing of a single right or left lung.

## Competing interests

The authors declare that they have no competing interests.

## Authors' contributions

MG designed the study, oversaw the biochemical analysis, participated in the calculation and presentation of the data, and wrote the draft of the manuscript. He is taking responsibility for the integrity of the work as a whole. JR has retrieved the data from the patients files, calculated and correlated the results, AS, AP, ASch and AH prepared the samples, performed cell sorting and in vitro work, as well as the biochemical analysis, PiL and PeL did extensive genetic analysis, FB did all pathology work. BS, OM, CS, JG-P, TN, KR and MG were engaged in treating the patient, performing lavages, designing clinical interventions, discussing and putting the data together. All authors read and approved the final manuscript.

## Pre-publication history

The pre-publication history for this paper can be accessed here:

http://www.biomedcentral.com/1471-2431/11/72/prepub
